# The clinical usefulness of the CTS5 in the prediction of late distant recurrence in postmenopausal women with estrogen receptor-positive early breast cancer

**DOI:** 10.1007/s12282-020-01130-y

**Published:** 2020-06-29

**Authors:** Wakako Tajiri, Hideki Ijichi, Katsumi Takizawa, Yumiko Koi, Takanobu Masuda, Hiroki Ueo, Chinami Koga, Yoshiaki Nakamura, Kenichi Taguchi, Masahiro Okamoto, Eriko Tokunaga

**Affiliations:** 1 Department of Breast Oncology, National Hospital Organization Kyushu Cancer Center, 3-1-1 Notame, Minami-ku, Fukuoka, 811-1395 Japan; 2 Department of Pathology, National Hospital Organization Kyushu Cancer Center, 3-1-1 Notame, Minami-ku, Fukuoka, 811-1395 Japan

**Keywords:** Breast cancer, ER+, Endocrine therapy, Late distant recurrence, CTS5

## Abstract

**Background:**

Clinical Treatment Score post-5 years (CTS5) is a promising prognostic tool to evaluate late distant recurrence (DR) risk for breast cancer after 5-year adjuvant endocrine therapy.

**Patients and methods:**

Among 560 postmenopausal women with pathological stage I–III estrogen receptor-positive (ER+) primary breast cancer, 383 women who had received 5-year adjuvant endocrine therapy without any recurrence at 5 years after surgery were included in this study. The CTS5 was calculated for each patient using a previously published formula, and the patients were stratified by their CTS5 values into the low-, intermediate- and high-CTS5 risk groups.

**Results:**

According to the CTS5, 205 (53.5%), 106 (27.7%) and 72 (18.8%) patients were classified into the low-, intermediate-, and high-CTS5 risk groups, respectively. A higher ER expression level was significantly associated with the low CTS5. The increased administration of adjuvant chemotherapy was significantly associated with a high CTS5. The occurrence of DR was higher in the intermediate and high CTS5 groups than in the low CTS5 group. The DRFS in the low CTS5 risk group was significantly better than that in the intermediate- or high-risk groups. In the ER-high or HER2-negative (HER2−) group, the DRFS in the low-risk group was significantly better than that of the intermediate- or high-risk groups. However, in the low-ER or HER2-positive group, there was no significant difference in DRFS among the three risk groups.

**Conclusions:**

In postmenopausal women with ER+ breast cancer, low CTS5 was considered to be associated with a very low risk of late DR. Thus, extended endocrine therapy may be unnecessary for patients with low CTS5 scores. Extended endocrine therapy should be offered for patients with intermediate or high CTS5 scores, especially those with high-ER and HER2− breast cancer.

## Introduction

Breast cancer is one of the most common cancers worldwide [1]. The incidence and mortality of breast cancer have increased annually in Japan according to Center for Cancer Control and Information Services, National Cancer Center, Japan [2]. The incidence has ranked first among all cancer sites in women since the 1990s. In Japan, two distinct peaks were observed, in the population-adjusted age distribution of breast cancer patients, in the late 40s and early 60s, because breast cancer has become increasingly prevalent in older women [3, 4]. The annual rates of patients with ER-positive (ER+) breast tumors increased from 71.8% in 2004 to 79.7% in 2011 [3]. Endocrine therapy is a very important treatment for the patients with ER+ breast cancer. Classically, 5-year endocrine therapy has been considered the gold standard adjuvant treatment, and has improved the patient outcomes [5]. However, at least 20–25% of these patients experience breast cancer recurrence [6, 7]. In half of these recurrent cases, recurrence occurred at more than 5 years after the initial diagnosis [6]. Japanese women with ER+ breast cancer also often experience late recurrence [8]. Several recent clinical studies have suggested the effectiveness of administering endocrine therapy for an extended period, beyond 5 years, for improving the prognosis [9–12]. However, extended endocrine therapy is not suitable for all the patients with ER+ breast cancer. For breast oncologists, it is very important to appropriately determine the patients who require extended endocrine therapy. The guideline of American Society of Clinical Oncology (ASCO) now recommends extended adjuvant endocrine therapy up to a total of 10 years for node-positive patients. For node-negative patients, the indication of extended adjuvant endocrine therapy should be determined based on considerations of the risk of recurrence and tolerability [13, 14]. If clinicians were able to predict a woman’s risk of late recurrence, they could limit their recommendation of extended endocrine therapy to women who can be expected to benefit from it.

The Clinical Treatment Score post-5 years (CTS5) was developed to estimate residual risk of late distant recurrence (DR) after 5 years of endocrine treatment [15]. To create the CTS5 tool, a data set that included data from a total of 11,446 postmenopausal women treated for ER+ breast cancer, who had completed 5 years of adjuvant endocrine therapy without any distant recurrence, in the randomized ATAC (Arimidex, Tamoxifen, Alone or in Combination, *N* = 4735) and BIG 1-98 (*N* = 6711) trials, was used. The CTS5 is expected to be used to predict the risk of late DR and to select the patients for extended therapy.

In the present study, we retrospectively evaluated the clinical significance of the CTS5 score in predicting the risk of late DR, after 5-year adjuvant endocrine therapy without any recurrences, in postmenopausal women with ER+ early breast cancer who were treated in our own department.

## Patients and methods

### Patient population

**Fig. 1:**
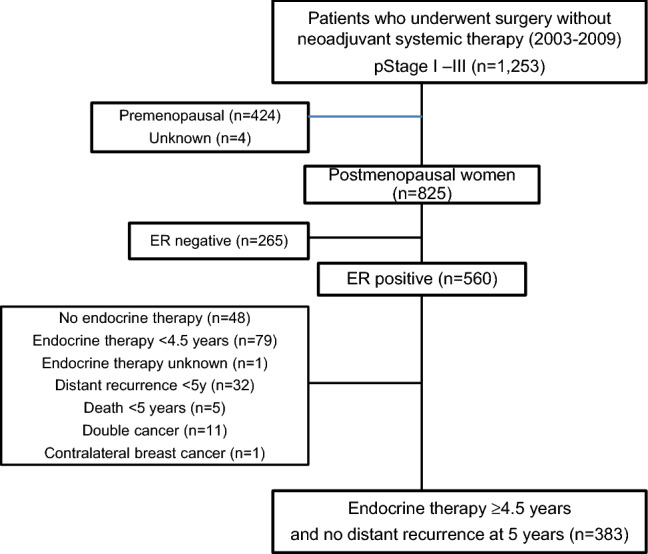
Schematic diagram of patient selection

A total of 1253 patients with pathological Stage I–III primary breast cancer, who underwent surgery without neoadjuvant systemic therapy in the Department of Breast Oncology, National Hospital Organization Kyushu Cancer Center between 2003 and 2009, were screened for the present study. Among them, 825 were postmenopausal women, and 560 had ER+ breast cancer. Finally, 383 postmenopausal women with ER+ breast cancer who had received 5-year (> 4.5 years) adjuvant endocrine therapy without any recurrence at 5 years after surgery were included in this study (Fig. 1). The clinical data were obtained from the patients’ medical records. Written informed consent was obtained from all patients before collecting the tissue samples. This study was approved by the institutional review board in our hospital.

### Pathological examination

All pathological examinations were performed by the experienced pathologists in our hospital. Specimens were regarded as ER- and PgR positive if the nuclear expression was ≥ 1%. The expression of ER and PgR was also evaluated using the Allred score [16]. In our study, the high expression of ER was defined by a total Allred Score (TS) of 7 or 8; while, the low expression of ER was defined by a TS of 3–6. The HER2 status was evaluated according to the recommendation of the ASCO/College of American Pathologists (CAP) [17].

### Calculation of CTS5

The CTS5 was calculated using the previously published formula [15]. This CTS5 model included age (continuous), tumor size (continuous), quadratic tumor size, nodal status (five groups: 0, negative; 1, one positive; 2, two to three positive; 3, four to nine positive; and 4, > nine positive), and grade (three groups: 1, low, grade 1; 2, intermediate, grade 2; and 3, high, grade 3). The CTS5 was calculated for each patient using the following formula [CTS5 = 0.438 × nodes + 0.988 × (0.093 × size − 0.001 × size^2^ + 0.375 × grade + 0.017 × age)], which is the final model induced by the combined ATAC and BIG 1-98 set [15]. The patients were stratified into three risk groups based on their calculated CTS5, which predicted their risk of DR; low risk (CTS5 < 3.13), intermediate risk (CTS5 3.13–3.86) and high risk (CTS5 > 3.86) according to this model.

### Statistical analyses

All statistical analyses were performed using the JMP software package (version 14.0; SAS Institute Inc., Cary, NC, USA). The associations between the clinicopathological characteristics were assessed using *χ*^2^ tests. Distant recurrence-free survival (DRFS) was defined as the time from the date of curative surgery to the detection of DR. We used the Kaplan–Meier method and Cox proportional hazards regression was used to perform the survival analysis. *P* values of < 0.05 were considered to indicate statistical significance.

## Results

### Patients’ characteristics

The characteristics of the patients included in this study are shown in Table 1. The median age was 61 years (range 42–87). More than 70% of the patients were classified as pathological T1 (pT1) and node negative, and histological grade 2 disease was the most prevalent. The rate of HER2-positivity was 11.4% and adjuvant chemotherapy was administered to 27.7% of patients. The high expression of ER was observed in 248 (64.8%) patients. According to the CTS5, 205 (53.5%), 106 (27.7%) and 72 (18.8%) patients were classified into the low-, intermediate-, and high-risk groups, respectively. The distributions of the risk categories are shown in Table 2. The patients with 4 or more positive lymph nodes were all categorized in the high-CTS5 group. In most patients in the low-CTS5 group, the tumor size was pT1, the nodes were negative and histological grade 3 was rarely observed.

**Table 1:** Clinicopathological characteristics of the patients

Factors	*n* (%)
Age (years)	
Mean (range)	61 (42–87)
Age groups (years)	
≤ 60	171 (44.6)
≥ 61	212 (55.4)
Tumor size (pathological T)	
pT1	280 (73.1)
pT2	96 (25.1)
pT3	7 (1.8)
Number of the positive nodes	
0	282 (73.6)
1	40 (10.4)
2, 3	30 (7.8)
4–9	25 (6.5)
≥ 10	6 (1.6)
Histological grade	
1	70 (18.3)
2	243 (63.4)
3	70 (18.3)
ER expression (TS)	
3–6	105 (27.4)
7–8	248 (64.8)
Unknown	30 (7.8)
PgR expression (TS)	
0–2	76 (19.8)
3–6	167 (43.6)
7–8	86 (22.5)
Unknown	54 (14.1)
HER2 status	
Negative	296 (86.6)
Positive	39 (11.4)
Unknown	4 (2.0)
Adjuvant endocrine therapy	
None	0
Administered	383 (100)
Administered endocrine therapy	
SERM	68 (17.8)
AI	230 (60.1)
SERM → AI	85 (22.2)
Adjuvant chemotherapy	
None	277 (72.3)
Administered	106 (27.7)
Distant recurrence	
None	364 (95.0)
5–10 years	12 (3.1)
> 10 years	7 (1.8)

*TS* total score of the Allred score, *SERM* selective estrogen receptor modulator, *AI* aromatase inhibitor

**Table 2:** Distributions of risk categories in each CTS5 risk group

Factors	No. (%)		
	Low	Intermediate	High
	205 (53.5)	106 (27.7)	72 (18.8)
Age, years mean (range)	61.5 (42–76)	62.6 (50–81)	63.9 (46–87)
Tumor size (pathological T)			
pT1	199 (71.1)	59 (21.1)	22 (7.9)
pT2	3 (3.1)	46 (47.9)	47 (49.0)
pT3	3 (42.9)	1 (14.3)	3 (42.9)
Number of the positive nodes			
0	200 (70.9)	73 (25.9)	9 (3.2)
1	4 (10.0)	25 (62.5)	11 (27.5)
2–3	1 (3.3)	8 (26.7)	21 (70.0)
4–9	0	0	25 (100.0)
≥ 10	0	0	6 (100.0)
Histological grade			
1	55 (78.6)	11 (15.7)	4 (5.7)
2	136 (56.0)	63 (25.9)	44 (18.1)
3	14 (20.0)	32 (45.7)	24 (34.3)

### Relationships between the CTS5 risk group and the clinicopathological characteristics

The relationships between the CTS5 and the clinicopathological characteristics are shown in Table 3. The higher ER expression level (*p* = 0.0113) and adjuvant endocrine therapy with selective estrogen receptor modulator (SERM) alone (*p* = 0.0387) were significantly associated with a low CTS5. An increased administration of adjuvant chemotherapy (*p* < 0.0001) was significantly associated with a high CTS5. On the other hand, there were no significant associations between the CTS5 risk groups and age, the expression of PgR or the HER2 status.

**Table 3:** Relationships between the risk groups classified according to the CTS5 and the clinicopathological characteristics

Factors	No. (%)			*P* value
	Low	Intermediate	High	
	205 (53.5)	106 (27.7)	72 (18.8)	
Age, years				
≤ 60	94 (55.0)	50 (29.2)	27 (15.8)	0.3864
≥ 61	111 (52.4)	56 (26.4)	45 (21.2)	
ER expression (TS)				
3–6	44 (41.9)	39 (37.1)	22 (21.0)	0.0113
7–8	149 (60.1)	57 (23.0)	42 (16.9)	
Unknown	12 (40.0)	10 (33.3)	8 (26.7)	
PgR expression (TS)				
0–2	36 (47.4)	23 (30.3)	17 (22.4)	0.4542
3–6	92 (55.1)	44 (26.3)	31 (18.6)	
7–8	53 (61.6)	21 (24.4)	12 (13.9)	
Unknown	24 (44.4)	18 (33.3)	12 (22.2)	
HER2 status				
Negative	167 (56.4)	73 (24.7)	56 (18.9)	0.1284
Positive	16 (48.7)	14 (35.9)	9 (23.1)	
Unknown	22 (45.8)	19 (39.6)	7 (14.6)	
Adjuvant endocrine therapy				
SERM	48 (70.6)	12 (17.6)	8 (11.8)	0.0387
AI	116 (50.4)	68 (29.6)	46 (20.0)	
SERM → AI	41 (48.2)	26 (30.6)	18 (21.2)	
Adjuvant chemotherapy				
None	190 (68.6)	65 (23.5)	22 (7.9)	< 0.0001
Administered	15 (38.7)	41 (38.7)	50 (47.2)	
Distant recurrence				
None	201 (55.2)	99 (27.2)	64 (17.6)	0.0353
5–10 years	3 (25.0)	4 (33.3)	5 (41.7)	
> 10 years	1 (14.3)	3 (42.9)	3 (42.9)	

*TS* total score of the Allred score, *SERM* selective estrogen receptor modulator, *AI* aromatase inhibitor

### Relationships between the CTS5 risk groups and the distant recurrences beyond 5 years after surgery

**Fig. 2:**
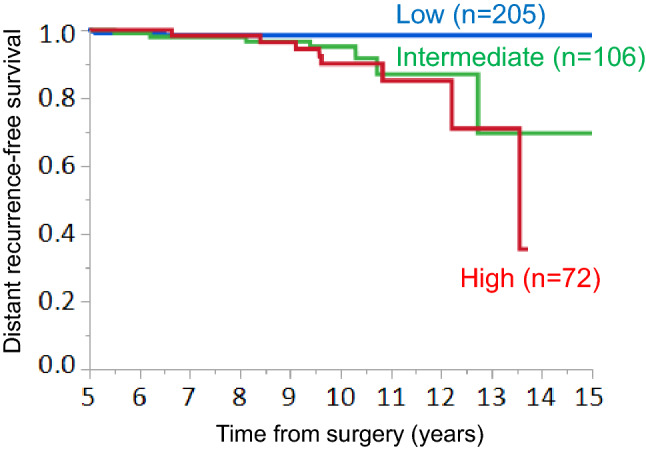
Relationships between the CTS5 risk category and distant recurrence-free survival (DRFS) beyond 5 years after surgery in the total cohort. DRFS in the three CTS5 risk groups. Low, *n* = 205; intermediate, *n* = 106; high, *n* = 72

The relationships between the CTS5 risk groups and DRFS beyond 5 years after surgery were analyzed. The median follow-up time was 9.9 (5.5–16.4) years. The occurrence of DR in the intermediate and high CTS5 groups was higher in comparison to the low CTS5 group (*p* = 0.0353). The incidence rates of DR at 5–10 years and later than 10 years after surgery in the low, intermediate and high CTS5 risk groups was 1.5% (*n* = 3) and 0.5% (*n* = 1), 3.8% (*n* = 4) and 2.8% (*n* = 3), 7.0% (*n* = 5) and 4.2% (*n* = 3), respectively (Table 3). The survival curves for DR are shown in Fig. 2. In the Cox regression analysis, the DRFS in the intermediate [Hazard ratio (HR) 4.33. 95% confidence interval (CI) 1.20–20.1, *p* = 0.0246] and high (HR 6.48. 95% CI 1.87–29.7, *p* = 0.0030) CTS5 groups were significantly poorer in comparison to the low CTS5 group (Table 4). On the other hand, there was no difference in the DRFS between the intermediate- and the high-CTS5 groups.

**Table 4:** Survival analysis for late DR after 5 years

Factors	Parameters CTS5 risk groups	HR	95% CI	*p* value
All patients	Intermediate vs. low	4.33	1.20–20.1	0.0246
	High vs. low	6.48	1.87–29.7	0.0030
	High vs. intermediate	1.50	0.53–4.28	0.4379
ER-high patients	Intermediate vs. low	14.7	2.48–278.9	0.0019
	High vs. low	13.6	2.00–265.4	0.0067
	High vs. intermediate	0.92	0.23–3.26	0.9006
ER-low patients	Intermediate vs. low	0.66	0.03–6.19	0.6598
	High vs. low	2.65	0.50–19.5	0.2513
	High vs. intermediate	4.50	0.63–89.8	0.1414
HER2− patients	Intermediate vs. low	5.17	1.10–36.2	0.0368
	High vs. low	7.58	1.74–51.9	0.0065
	High vs. intermediate	1.47	0.44–5.13	0.5288
HER2+ patients	Intermediate vs. low	3.11	0–6.66	0.2622
	High vs. low	3.11	13.3–13.3	0.3942
	High vs. intermediate	1	11.98–	1

*p* value generated by a univariate Cox analysis

*DR* distant recurrence, *HR* hazard ratio, *CI* confidence interval

### Relationships between the CTS5 risk groups and distant recurrences according to the expression of ER and the HER2 status

**Fig. 3:**
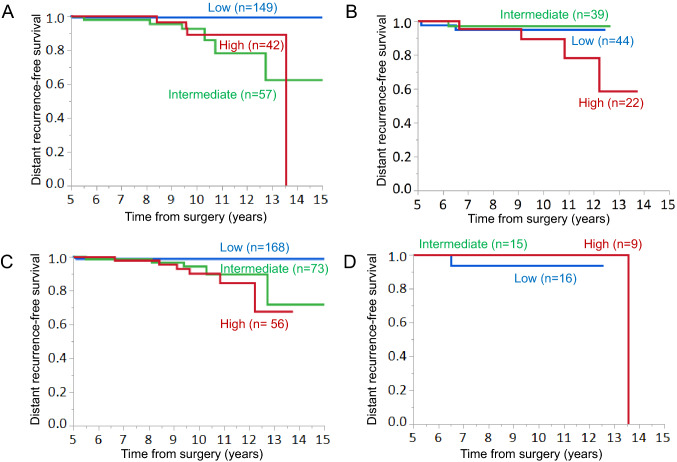
The relationships between the CTS5 risk categories and distant recurrence-free survival (DRFS) beyond 5 years after surgery according to the ER expression and HER2 status. **a** DRFS in the three CTS5 risk groups in the ER-high group. CTS5 low, *n* = 149; intermediate, *n* = 57; high, *n* = 42. **b** DRFS in the three CTS5 risk groups in the ER-low group. CTS5 low, *n* = 44; intermediate, *n* = 39; high, *n* = 22. **c** DRFS in the three CTS5 risk groups in the HER2− group. CTS5 low, *n* = 168; intermediate, *n* = 73; high, *n* = 56. **d** DRFS in the three CTS5 risk groups in the HER2+ group. CTS5 low, *n* = 16; intermediate, *n* = 15; high, *n* = 9

The ER expression level and HER2 status have some impact on the prognosis and the sensitivity to the endocrine therapy. We, therefore, analyzed the relationships between the CTS5 risk groups and DRFS according to the ER expression level and the HER2 status (Fig. 3; Table 4). In the ER-high patients, the DRFS in the intermediate (HR 14.7, 95% CI 2.48–278.9, *p* = 0.0019)- and high (HR 13.6, 95% CI 2.00–265.4, *p* = 0.0067)-CTS5 groups were significantly poorer in comparison to the low-CTS5 group (Fig. 3a; Table 4). In the HER2-negative (HER2−) patients, the DRFS in the intermediate (HR 5.17, 95% CI 1.10–36.2, *p* = 0.0368)- and high (HR 7.58, 95% CI 1.74–51.9, *p* = 0.0065)-CTS5 groups were significantly poorer in comparison to the low-CTS5 group (Fig. 3c; Table 4). There was no difference in the DRFS between the intermediate and the high-CTS5 groups in the ER-high and HER2− patients. However, the DRFS did not differ according to the three CTS5 risk groups in the ER-low and HER2+ patients (Fig. 3b, d; Table 4).

## Discussion

Adjuvant endocrine therapy has improved the outcomes of women with hormone receptor-positive early breast cancer, however, breast-cancer recurrences continue to occur steadily after 5 years of adjuvant endocrine therapy [5, 7]. Recent studies showed the effectiveness of extended endocrine therapy for reducing the breast cancer recurrence in comparison to the conventional 5-year treatment, although the absolute benefits of the extension are modest [9–12]. In an ASCO guideline, extended endocrine therapy for up to a total of 10 years, including AIs, is recommended for women with node-positive breast cancer [13]. For women with node-negative breast cancer, the indication of extended adjuvant endocrine therapy is determined based on the considerations of the balance of benefits and harm, the reduction of the risk of recurrence and the side effects or costs associated with endocrine therapy.

The CTS5 is a recently reported tool that is used to predict late DR after 5-year adjuvant endocrine therapy [15]. Dowsett et al. reported that CTS5 was able to accurately separate the women into three groups of low (risk of late distant recurrence less than 5%), intermediate (risk between 5 and 10%) and high (risk more than 10%) risk of late DR after 5 years of endocrine therapy [15]. In the present study, the incidence of DR in the low-CTS5 risk group was significantly lower than that in the intermediate- and high-CTS5 risk groups. Actually, the women in this low-CTS5 risk group experienced no DR in our dataset. However, we could not find any significant differences in late DR between the intermediate- and high-risk groups categorized according to the CTS5. There might be differences in the patient’ backgrounds between the patients enrolled into the ATAC or the BIG 1-98 trials and those treated in our hospital. In the study of the CTS5 with the data of ATAC and the BIG 1-98 study, the estimated 5–10-year DR risk in the low-, intermediate- and high-CTS5 risk groups was 2.5% (ATAC) and 3.6% (BIG 1-98), 6.9% (ATAC) and 7.7% (BIG 1-98), and 17.3% (ATAC) and 20.3% (BIG 1-98), respectively. In contrast, in our cohort, the incidence of DR in 5–10 years in the low-, intermediate- and high-CTS5 risk groups was 3 (1.5%), 4 (3.8%) and 5 (7.0%), respectively. It seems that the incidence of DR in our cohort was markedly lower in comparison to the cohorts of the ATAC and the BIG 1-98 trial. These factors are considered to have been responsible for the observation that there was no difference in late DR between the intermediate- and high-CTS5 risk groups. Thus far, one group reported the validation of the CTS5 model in the breast cancer population [18]. The study included a total of 23,168 patients whose data were registered in the Surveillance, Epidemiology, and End Results (SEER) database. The authors of this study noted the usefulness of the CTS5 for evaluating the late recurrence risk. However, the outcomes that were evaluated in the study were overall survival (OS) and breast cancer-specific survival (BCSS), and not the DR. In addition, the median follow-up time was only 65 months, which seems too short to evaluate late recurrence beyond 5 years (60 months).

We paid close attention to the relationships between the ER expression level and HER2 status and the impact of CTS5 on late DR, because the ER expression level and HER2 status affect the sensitivity to endocrine therapy [19, 20]. Regarding the ER expression level, tumors in which 1–100% of the nuclei are positive for ER should be regarded as ER-positive according to ASCO/CAP ER and PgR testing guideline [21]. However, many breast oncologists acknowledge that there are limited data on the benefit of endocrine therapy in patients with tumors in which 1–10% of the nuclei are ER positive. In the latest ASCO/CAP guideline, a new reporting category, “ER Low Positive” is recommended for cancers in which 1–10% of the cells are ER positive [22]. A majority of low-ER breast cancers behave like hormone receptor-negative tumors or triple-negative breast cancer (TNBC), and the sensitivity to the endocrine therapy is poor [23, 24]. TNBC survivors who have been disease free for 5 years have a low probability of experiencing recurrence over the subsequent 10 years [25]. These characteristics of the low-ER breast cancer support our finding that the DRFS did not differ according to the CTS5 risk group in ER-low patients. In the present study, in the ER-high patients, the DRFS in the intermediate- and high-CTS5 groups was significantly poorer in comparison to the low-CTS5 group. These findings are compatible with previous reports that demonstrated that the tumors with the high expression of estrogen-responsive genes or tumors with the expression of genes associated with the highly proliferative/high-ER activity had a higher risk of late recurrence [26, 27]. In terms of the HER2 status, the DRFS in the intermediate- and high-CTS5 groups was significantly poorer in comparison to the low-CTS5 group in HER2− patients, but not in HER2+ patients. The previous study showed that distant metastasis of the HR+/HER2+ subtype often occurred at 2–5 years during follow-up, while distant metastasis of the HR+/HER2− subtype gradually increased after 5 years of follow-up, although the incidence was significantly lower in comparison to the HR+/HER2+ subtype [28]. This report supports our finding that there were no significant differences in late DR among the three CTS5 risk groups in HER2+ patients.

Multigene assays have been investigated as predictors of late recurrence (reviewed by Bense et al.) [29]. Prosigna, Breast Cancer Index and EndoPredict/EPclin could predict the late DR risk, but not Oncotype Dx in the subset of the TransATAC trial [30–33]. These previous studies provided a great deal of important information; however, those multigene tests are not always available in the ordinary practice. In Japan, multigene assays are not covered by medical insurance. In comparison to these multigene assays, CTS5 is highly clinically useful, because it is calculated from clinicopathological factors that are always available and the CTS5 calculator is available online for free.

The strength of this study is that these data are from a single institution and that the follow-up time was significantly long, with high-quality follow-up and updated clinical data. However, the present study is also associated with several limitations. All data are retrospective, the sample size was relatively small, and the number of events for DR was also relatively small, which made it difficult to draw a distinctive conclusion.

In conclusion, based on the results obtained from our analysis in postmenopausal women with ER+ breast cancer, tumors with a low CTS5 risk score have a very low risk of late DR; while, intermediate- and high CTS5 risk scores were associated with late DR after 5-year adjuvant endocrine therapy. The extension of endocrine therapy is considered to be unnecessary for patients with low CTS5 scores. For patients with intermediate or high CTS5 scores, extended endocrine therapy should be offered, especially in the patients with high-ER and HER2− breast cancer.
